# Iodinated Contrast Media—From Clinical Use to Environmental Concern and Treatment Possibilities

**DOI:** 10.3390/molecules31030551

**Published:** 2026-02-04

**Authors:** Katarzyna Wrzesińska, Michał Kwiatkowski, Piotr Terebun, Dawid Zarzeczny, Agata Sumara, Tomoyuki Murakami, Nobuya Hayashi, Frantisek Krcma, Evgenia Benova, Karol Hensel, Zdenko Machala, Emilia Fornal, Joanna Pawłat

**Affiliations:** 1 Department of Electrical Engineering and Smart Technologies, Lublin University of Technology, Nadbystrzycka Street 38A, 20-618 Lublin, Poland; d589@pollub.edu.pl (K.W.);; 2 Department of Bioanalytics, Medical University in Lublin, ul. Jaczewskiego 8b, 20-090 Lublin, Poland; 3 Faculty of Science and Technology, Seikei University, 3-3-1 Kichijoji-Kitamachi, Musashino 180-8633, Japan; 4 Interdisciplinary Graduate School of Engineering Sciences, Kyushu University, Fukuoka 816-8580, Japan; 5 Institute of Physical and Applied Chemistry, Brno University of Technology, Purkyňova, 464/118, 612 00 Brno, Czech Republic; 6 Center of Competence “Clean Technologies for Sustainable Environment—Water, Waste, Energy for Circular Economy”, Sofia University “St. Kliment Ohridski”, 1164 Sofia, Bulgaria; 7 Faculty of Mathematics, Physics and Informatics, Comenius University, 842 48 Bratislava, Slovakia

**Keywords:** iodinated contrast media, environmental impact, disinfection by-products, AOP

## Abstract

Iodine-based contrast agents (ICMs) are crucial substances in medical imaging because of their potent X-ray characteristics and chemical stability. However, their persistence and poor removal in conventional wastewater treatment have led to increasing environmental concern. Although ICMs exhibit low acute toxicity, their transformation during water disinfection can generate iodine-based disinfection by-products (I-DBPs), like iodo-trihalomethanes, which display notable cytotoxic, genotoxic, and ecotoxic effects and compromise drinking water quality. Advanced oxidation processes (AOPs) have become promising methods for breaking down persistent ICMs and limiting the formation of I-DBPs. Techniques including ozonation, UV/H_2_O_2_, UV/chlorine, photocatalysis with TiO_2_, Fenton reactions, and electrochemical oxidation utilize highly reactive radicals to decompose persistent compounds like iopamidol, iohexol, iopromide, and diatrizoate. Despite high degradation efficiencies under laboratory conditions, limitations such as incomplete mineralization, secondary product formation, and elevated operational costs hinder large-scale implementation. Future research should focus on optimizing AOP conditions under realistic water matrices, evaluating by-product toxicity, and developing cost-effective hybrid systems. Advancing these technologies is critical to reducing the environmental burden of ICMs and safeguarding aquatic ecosystems and public health.

## 1. Introduction

Growing population numbers and the evolution of diverse industrial sectors necessitate a significant supply of water with specific, controlled parameters [1]. Undoubtedly, this poses a huge challenge to current water management. Every year, 380 billion m^3^ of wastewater is produced, compared to current levels, it is believed that wastewater production will increase by 24% by 2030 and 51% by 2050 [2]. Based on available literature data [3], pharmaceuticals are the main water pollutants. Each year, the consumption of active pharmaceutical ingredients exceeds 100,000 tons; this group includes, for example, antibiotics, painkillers, lipid regulators, β-blockers and iodinated contrast media. Substances that are consistently detected in wastewater are iodinated contrast agents [4]. The identification of X-rays by Wilhelm C. Röntgen in 1895 marked the beginning of extensive progress in diagnostic imaging [5]. It is a medical discipline that enables the visualization of the human body without the need for surgical intervention. Radiology techniques such as X-ray, CT, X-ray, MRI or CAT scan are most commonly used to perform such examinations. In order to improve the obtainable image of specific body structures, contrast agents are used. Contrast agents are categorized as diagnostic drugs that significantly improve the visibility of examined tissues, organs and increase the chances of seeing additional details [6,7]. Therefore, ICMs are the most frequently utilized pharmaceuticals in diagnostic imaging. These substances have a benzene ring framework with attached iodine atoms. Iodine is responsible for stronger X-ray absorption, which amplifies the brightness of structures containing the contrast agents in the image. ICMs are constantly being improved to prevent undesirable toxicological effects during X-ray examinations [8,9]. They exhibit extremely high chemical and biological stability. However, residues of these substances and their TPs at μg/L levels have been identified in aquatic systems. The continuously rising human use of pharmaceuticals is driving higher concentrations of ICMs in the environment [10]. ICMs are drawing growing scientific attention because of the elevated risk of generating toxic iodinated byproducts (I-BPs) during water treatment processes [11]. According to literature reports, toxicity studies on aquatic organisms, i.e., bacteria or plants, have not shown toxic effects of ICMs, but iodinated compounds have been the primary sources of elevated organic iodine (AOI) levels in hospital effluents [5]. In addition, iodinated byproducts (I-DBPs) can be formed during the water disinfection process. These compounds exhibit cytotoxic, ecotoxic and genotoxic effects [6,7].

## 2. ICM

### 2.1. Development of Iodinated Contrast Agents

In 1918, silver was replaced in urological tests with solutions of potassium iodide and sodium iodide. These substances seemed to be significantly better in terms of, among others, stability and low price. Sodium iodide was also used in radiology to visualize the urinary system, and it also allowed for the diagnosis of diseases such as atherosclerosis [12]. However, it contributed to an increase in blood osmolarity due to dissociation in aqueous solutions, which caused severe pain in patients [13]. In the 1930s, the first iodinated contrast agent was introduced—sodium 2-oxy-5-iodo-pyridine-N-acetate, commercially known as Uroselectan. This compound was characterized by the presence of covalent bonds and was much less toxic than sodium iodide. However, it was quite quickly replaced by iodopiracet (Diodrast), a compound characterized by better solubility and radiological contrast [14]. This drug caused serious side effects in patients, including nerve cell toxicity. In the 1950s, the first benzene–based contrast agent appeared—sodium acetrizoate (Urokon). The benzene ring was selected because it could carry more iodine atoms and hydrophilic functional groups, which resulted in lower osmolality. Urocon was more opaque to X-rays, but, like Diodrast, it contributed to neurotoxicity. For this reason, further contrast agents have appeared, based on triiodobenzenic acid. The main examples of such compounds are diatrose acid and metrizoate. These compounds are composed of 2,4,6-triiodobenzeic acid, in which the iodine atoms are arranged symmetrically in the ratio of hydrophilic groups, which influence the solubility in water. In 1960, triiodobenzoic compounds appeared in which the carboxyl group was replaced by an amino group. These compounds were primarily non-ionic, which alleviated side effects and also contributed to a decrease in their osmolality. Subsequently, further non-ionic contrast agents were introduced [15]. These included iopromide, iopamido, ioversol and iohexol. In the 1990s, hexaiodinated dimers were introduced, both ionic—ioxaglate and non-ionic—iotrolan, iodixanol. These compounds have two benzene rings with symmetrically arranged hydroxyl groups [16]. However, research on the safety of these compounds is presented in a controversial manner. In summary, the ideal contrast agent should be low–toxicity molecules including high iodine content to attenuate large amounts of X-ray radiation [17]. Additionally, these compounds should have low viscosity so that they can be easily administered to patients. It should be taken into account that these guidelines may be associated with an increase in blood osmolality [18].

### 2.2. Structure of Iodine Contrast Media

**Figure 1:**
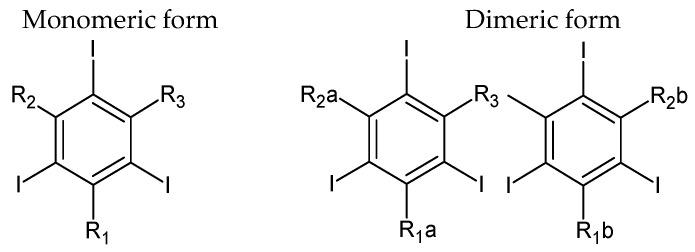
Molecular structural units of ICM [18].

These contrast agents for X-rays are based on 2,4,6-triiodobenzoic acid, and the majority feature a triiodine-substituted benzene ring (Figure 1). Iodine is crucial for X-ray attenuation because of its atomic radius, which is approximately 133 picometers. However, the wavelength of X-ray radiation covers the range of 10–10,000 picometers.

Iodine atoms arranged in the benzene ring increase the size of the molecule, causing X-ray attenuation. Additionally, the covalent bond with the organic functional group significantly lowers the potential toxicity of free iodide. Moreover, formation of monomers and dimers is possible for these compounds. Monomers contain a single triiodinated benzene ring, whereas dimers comprise two such rings linked together and attached to an additional functional group. Figure 1 shows the ionic carboxylate group (R_1_, R_1_a, R_1_b) and the lack of a carboxylate group (R_2_, R_2_a, R_2_b, R_3_) located in the side chain. On this basis, four basic classes of ICM are distinguished: (1) a nonionic monomer consisting of a single triiodinated benzene ring, lacking both a carboxylate substituent and any additional benzene substituent, (2) an ionic monomer containing one triiodinated benzene ring with a carboxylate substituent attached to the benzene ring, (3) a nonionic dimer composed of two triiodinated benzene rings, without any carboxyl functional groups, (4) an ionic dimer formed from two linked triiodinated benzene rings, in which at least one benzene ring carries a carboxylate-containing substituent. ICMs have various properties (Table 1), which affects their use for specific indications. Additionally, ionic monomers should be administered in very high concentrations due to their high osmolality in relation to blood. Therefore, they have a lower ability to attenuate X-ray radiation. Non-ionic monomers as well as ionic dimers are characterized by a much lower osmolality range. Some of them are iso-osmolar with blood [19].

### 2.3. Application of Iodine Contrast Media

Radiological examinations often use contrast agents designed to increase tissue resolution. In computed tomography angiography and classic X-ray angiography, it is possible to visualize the lumen of vessels, their dilatation, narrowing or closure. Contrast agents make it possible to assess the vascularization of lesions and thus approximate the nature of a given lesion. They are also useful for demonstrating the function of organs, for example, in urography, where we can observe the process of excretion of the contrast agent by the kidneys. Another important property is to reproduce the appearance of the walls of anatomical structures, which allows us to diagnose fistulas, diverticula, assess the tightness of anastomoses after surgical operations or visualize a foreign body stuck in the esophagus [20]. Positive and negative contrast agents are used during X-ray examinations. Positive contrast agents increase radiation absorption and “brighten” anatomical structures on radiographic images. Positive contrast agents used in daily clinical practice include barium sulfate and iodine contrast agents. Barium sulfate is a chemical compound that is insoluble in water, while iodine contrast agents are water-soluble. Negative contrast agents reduce the absorption of X-rays and, as a result, “darken” the area under examination where they are present [21]. Examples of negative contrast agents include gases like molecular oxygen, carbon dioxide or air, and water. In magnetic resonance imaging, there is also a division between positive and negative contrast agents, but this is due to the magnetic properties of substances that have unpaired electrons. In gastrointestinal diagnostics, the patient drinks or receives barium sulfate rectally by infusion, and iodine contrast agent if there are clinical contraindications. Iodine contrast agents, in addition to the gastrointestinal tract, can be administered into the urinary tract, genital tract, biliary tract, spinal canal, joint cavity, intravenously and intraperitoneally. In magnetic resonance imaging, preparations are used orally, intravenously or intrathecally. Agents used in ultrasound are applied only intravenously. Barium sulfate is not absorbed from the gastrointestinal tract and is excreted unchanged in the feces. Iodine contrast agents are excreted mainly through the kidneys and to a lesser extent through the liver with bile. Ultrasound agents are removed from the body through the lungs with exhaled air [22].

### 2.4. The Impact of ICM on the Human Body

**Figure 2:**
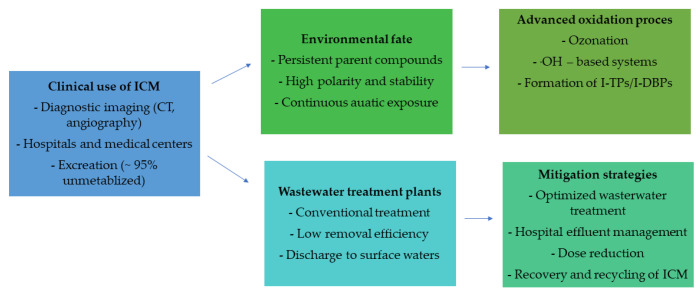
Pathways, degradation, and mitigation of iodinated contrast media from clinical use to the aquatic environment.

Iodine contrast agents are considered one of the safest medical drugs. Unfortunately, like any medical agent, they can cause adverse reactions due to hypersensitivity and their toxic effects. In addition, they can interact with other drugs taken by patients with oncological diseases. The percentage of the incidence of hypersensitivity symptoms after the use of ionic contrast agents is relatively small; moreover, the number is decreasing with the introduction of new low-ionic medical agents. Despite this, the number of such events is steadily increasing with the increasing importance of modern interventional and contrast agent imaging techniques. This is determined by the high number of 75 million radiological examinations with these agents in a single year worldwide. In addition, the ever-increasing threat of hypersensitivity reactions associated with the indiscriminate use of contrast agents prompts us to expand our knowledge of the principles of diagnostic management of hypersensitivity to the medical agents used [23,24]. Symptoms of hypersensitivity to iodine contrast media can occur at any age regardless of gender. A study involving 220 people with hypersensitivity to ICM, found that hypersensitivity symptoms occurred in people ranging from 12 to 83 years old, with a median age of 54. However, the data obtained are related to the higher frequency of diagnostic imaging within a certain age range rather than a predisposition to hypersensitivity. In addition, it was found that a higher number of disclosed reactions occurred after administration of contrast agent intravenously than intraarterially, this is related to the fact that medical indications much more often require intravenous contrast. So far, the studies conducted have not proven that food allergy and atopy to iodine-rich foods such as seafood increase the chances of hypersensitivity symptoms to ICM. However, some studies show a higher risk of hypersensitivity symptoms in patients with respiratory allergies [25,26]. Symptoms of hypersensitivity to iodine contrast agents are divided according to the timing of the reaction. Clinical reactions of the body caused by iodine contrast agents are differentiated into immediate reactions, i.e., those that appear within 1 h, and non-immediate reactions, manifesting up to 7 days after application of the medical preparation. The first type of reaction to iodine contrast agents usually reveals itself between 15 and 60 min after the contrast agent is administered. The main symptoms are angioedema, urticaria, shortness of breath and vomiting. Mostly, the aggravation of these symptoms is mild and lasts a short time. Sometimes, however, anaphylactic reactions occur, and in extreme cases even fatal. Symptoms of immediate hypersensitivity can occur in people not previously exposed to iodine contrast agents, in patients already exposed to such preparations despite the fact that previous contrast studies did not cause such a reaction in the body, as well as in people with a history of hypersensitivity symptoms after contrast application. The correlations presented here come from the results of the Brockow study, which involved 122 patients with immediate reactions to iodine contrast agents. The study found that hypersensitivity symptoms occurred in 69 patients who had never undergone examinations with iodine contrast and in 53 patients who underwent diagnostic imaging with this type of preparation again. Symptoms of immediate hypersensitivity reactions to iodine contrast agents are much more severe and frequent after high-osmolality ionic preparations than non-ionic low-osmolality ones. Antihistamines or glucocorticosteroids are recommended for patients at risk of recurrence of immediate response to contrast media. However, adequate data on the efficacy of such treatment are still lacking [27,28]. The second type of reaction is non-immediate hypersensitivity symptoms to iodine contrast. The body’s reactions usually appear within 48 h. However, the period of their occurrence can range from 1 h to 7 days. The main symptoms are dermatological complaints, for example, urticaria or toxic epidermal necrolysis. In people with non-immediate reactions, reapplication of iodine contrast agents is much less likely to cause allergic symptoms than immediate reactions. Brockow, in his study, found a re-reaction in only 25% of patients, in addition, the clinical symptoms that occurred were of similar severity. Mostly, these symptoms are caused by nonionic dimeric agents, although some researchers claim that the incidence of symptoms is not related to the osmolality, ionization of the drug or iodine content. Regarding non-immediate reactions, there is no basis for prophylaxis in the form of antihistamines or glucocorticosteroids [29]. General safety precautions for the use of iodine contrast agents include, first and foremost, a properly trained physician who will respond quickly in the event of an adverse reaction. Those who are authorized to administer such preparations should be knowledgeable about severe clinical symptoms. In addition, every patient should remain under the care of a physician for the first five minutes after applying iodine contrast. All reactions that occur after the administration of such a preparation should be documented so that they can be prevented in the future [30]. The main pathways, degradation processes, and mitigation strategies related to iodinated contrast media are summarized in Figure 2.

The most widely used iodinated contrast media include non-ionic monomeric and dimeric compounds such as iohexol, iopamidol, iopromide, iomeprol. These agents are administered in large quantities during contrast-enhanced diagnostic procedures and are excreted by patients predominantly in unchanged form within 24 h after administration. Consequently, hospitals and diagnostic centers constitute point sources of ICM emissions, with municipal wastewater treatment plants (WWTPs) acting as the main gateways for their release into the aquatic environment. Due to their high polarity, low volatility, and resistance to biodegradation, ICM are poorly removed during conventional wastewater treatment processes. Numerous monitoring studies have reported the presence of ICM in WWTP effluents, surface waters, groundwater, and even drinking water sources at concentrations ranging from ng L^−1^ to low µg L^−1^ levels. Their continuous discharge leads to pseudo-persistent behavior in aquatic ecosystems, despite relatively low individual environmental concentrations, raising concerns about long-term exposure of aquatic organisms and potential accumulation in water resources [20,31,32]. Although iodinated contrast media are generally considered to exhibit low acute toxicity, their environmental relevance arises from chronic exposure, mixture effects, and the formation of transformation products. Ecotoxicological studies indicate that parent ICM typically show limited short-term toxicity toward algae, invertebrates, and fish; however, sublethal effects such as oxidative stress, altered enzyme activity, and endocrine-related responses have been reported at environmentally relevant concentrations. Particular concern is associated with the formation of iodinated transformation products and iodinated disinfection by-products (I-DBPs), which may exhibit significantly higher toxicity than their parent compounds. Iodinated by-products formed during water disinfection processes have been shown to possess enhanced cytotoxicity and genotoxicity compared to chlorinated and brominated analogues. These findings suggest that the environmental hazard of ICM cannot be assessed solely based on the toxicity of the parent compounds but must also consider the toxicity of their degradation products [33,34]. In natural aquatic environments, iodinated contrast agents are characterized by high chemical stability and resistance to biological degradation. Conventional environmental processes such as hydrolysis and biodegradation contribute only marginally to their removal. Photodegradation may occur under specific conditions; however, its overall contribution to ICM attenuation in surface waters is limited due to their low absorption in the environmentally relevant UV–visible range. As a result, ICM exhibit long environmental half-lives and are frequently detected far from their original emission sources. Their persistence facilitates continuous transport within aquatic systems, including infiltration into groundwater and potential entry into drinking water treatment chains, thereby extending their environmental and human exposure pathways [35]. Conventional wastewater treatment processes, including activated sludge systems, typically achieve low removal efficiencies for ICM, often below 20%. Their physicochemical properties hinder adsorption onto sludge and limit biodegradation, resulting in inefficient elimination during standard treatment stages. Advanced oxidation processes (AOPs), such as ozonation, UV/H_2_O_2_, and hydroxyl radical-based systems, have been extensively studied as effective methods for ICM degradation. These processes can substantially reduce parent compound concentrations; however, they frequently lead to the formation of intermediate transformation products, including iodinated organic compounds and inorganic iodine species. During ozonation and UV-based treatments, the cleavage of C–I bonds may result in the release of iodide, which can further participate in the formation of iodinated disinfection by-products during subsequent chlorination or chloramination steps. Therefore, while AOPs represent a promising approach for the removal of ICM from wastewater and drinking water, their application requires careful optimization and comprehensive assessment of transformation pathways and by-product toxicity to avoid unintended environmental consequences [36].

### 2.5. Iodine Contrast Media in the Environment

In the 1990s, the annual use of iodinated contrast agents was 3.5 × 10^6^ kg due to their intravenous administration in very high doses. 75% of ICM is excreted from the human body within 4 h in urine into hospital sewage [35,37]. However, within 24 h, practically 100% of the administered dose is achieved. Iodinated contrast agents are the primary contributors to elevated levels of organic halogens (AOX) found in hospital wastewater [38]. The problem is that hospital sewage is not selectively treated and ends up in the local sewage system [39]. Polar pollutants readily migrate into surface waters, where they can negatively affect both aquatic life and human health. ICMs contain an amino functional group, which promotes the formation of nitrogenous by-products, e.g., haloacetamide or haloacetonitrile, which exhibit significant toxicity [40,41]. Additionally, water disinfection processes may produce iodized trihalomethane, which is also a toxic by–product of ICM. A global upward trend in the environmental occurrence of iodinated contrast media (ICM) has been reported worldwide, reflecting their increasing use in diagnostic imaging and their continuous release into wastewater systems [42,43]. Municipal wastewater treatment plants represent the primary pathway for ICM diffusion into the aquatic environment, as these compounds are excreted largely unmetabolized and are only poorly removed during conventional treatment processes. As a result, ICM are frequently detected in hospital effluents, wastewater treatment plant effluents, surface waters, and downstream drinking water resources [44]. The most commonly reported iodinated contrast agents in the environment include iopromide, iohexol, iopamidol, iomeprol, and diatrizoic acid. Several of these compounds have been monitored for nearly two decades, highlighting their environmental relevance and persistence in aquatic systems. Long-term monitoring data from the RIWA-Rijn water quality program, coordinated by the Dutch association of drinking water suppliers, indicate the continuous presence of iohexol, iopamidol, iopromide, iomeprol, and diatrizoic acid in the Rhine River since 2002. In 2020, the daily load of iodinated contrast agents at the Rhine monitoring station in Lobith ranged from 10.3 to 175 kg day^−1^, with the cumulative annual load in the Germany–Netherlands border region exceeding 70 tons. Although iodinated contrast media are generally characterized by relatively low acute toxicity, their environmental significance arises from their continuous discharge, long-term exposure, and limited removal during wastewater treatment. The persistence of ICM in treatment systems may also promote the formation of potentially harmful transformation products during conventional and advanced treatment processes [44]. Trend analyses between 2010 and 2019 revealed a gradual annual decrease in diatrizoic acid concentrations (approximately 2.7%), likely associated with its phased-out clinical use and replacement by alternative contrast agents or barium sulfate-based formulations. In contrast, concentrations of iopromide and iohexol increased annually by 2.4% and 3.1%, respectively, while no significant temporal trends were observed for iopamidol and iomeprol. These findings emphasize the need for continuous environmental monitoring and the timely inclusion of newly introduced contrast agents in drinking water quality surveillance programs [34].

The environmental occurrence and persistence of iodinated contrast media are strongly governed by their physicochemical properties. Most clinically used ICM are characterized by high water solubility, pronounced polarity, and low volatility, which facilitate their rapid transport within aquatic systems and limit their removal by conventional wastewater treatment processes. Their molecular structures, typically containing multiple iodine atoms bound to aromatic rings, confer high chemical stability and resistance to hydrolysis, photolysis, and biodegradation under environmentally relevant conditions. As a consequence, ICM exhibit limited sorption to suspended solids and activated sludge, resulting in poor elimination during primary and secondary treatment stages. These properties explain the frequent detection of ICM in wastewater effluents and surface waters at concentrations reported in Table 2. Furthermore, the stability of the carbon–iodine bond hinders complete mineralization, favoring persistence in the environment and potential transformation into iodinated by-products during advanced treatment or disinfection processes. Overall, the combination of high solubility, chemical stability, and resistance to biological degradation renders iodinated contrast media pseudo-persistent micropollutants, emphasizing the need for advanced treatment technologies and targeted management strategies to reduce their environmental release [20,44,45]. The observed reduction in ICM levels could result from the use of a newly introduced compound. Due to their physicochemical properties, iodinated contrast agents represent a risk to drinking water quality. They are characterized by metabolic stability and high water solubility, which significantly complicates their removal during drinking water purification. Water treatment relies on conventional methods such as flocculation and sedimentation to remove suspended particulates, sand filtration to facilitate microbial degradation, and activated carbon adsorption to address taste, color, and odor. However, such methods cannot completely remove iodinated contrast agents. If increased levels of contaminants increase the required purification volume, additional processes are implemented. The most common are advanced oxidation techniques, like the integration of ultraviolet radiation with hydrogen peroxide, or ozonation.

Although advanced treatment techniques are highly effective in removing iodinated contrast media, their physicochemical properties strongly influence both degradation efficiency and the formation of transformation products [34]. The high water solubility and pronounced polarity of most iodinated contrast agents facilitate their transport in aqueous systems but limit their sorption onto biomass and suspended solids, thereby reducing removal during conventional treatment processes. At the same time, their chemical stability—particularly the presence of multiple iodine atoms bound to aromatic structures—hinders complete mineralization and promotes partial transformation rather than full degradation. As a result, advanced oxidation processes may lead to the formation of iodinated transformation products, some of which may exhibit enhanced reactivity or toxicity compared to the parent compounds [45,48]. In addition to abiotic processes, certain iodinated contrast agents are susceptible to biotransformation under both anaerobic and aerobic conditions, which is particularly relevant for drinking water purification and natural attenuation processes. Iopromide represents a well-documented example, exhibiting a complex transformation pathway that includes both anaerobic and aerobic metabolites. Reported transformation mechanisms include anilide hydrolysis and deiodination under both redox conditions, as well as oxidation of primary and secondary hydroxyl groups, oxidative decarboxylation, cleavage of the amide–methyl bond, dehydroxylation, and deacetylation occurring predominantly under aerobic conditions. These findings demonstrate that the environmental fate of iodinated contrast media is governed by an interplay between their intrinsic chemical stability, high solubility, and the redox conditions prevailing during wastewater treatment and environmental processes [33,35]. However, complete mineralization to yield elemental iodine has not yet been accomplished. Water utilities utilize chlorine or chloramine to guarantee microbial stability and disinfection throughout water transport. The use of chlorine is strictly controlled because it can produce disinfection by-products. Chloramine is favored owing to its reduced reactivity with dissolved organic matter; nevertheless, its interaction with iodinated contrast media can result in the formation of iodinated DBPs. Published studies suggest that these compounds exhibit higher cytotoxicity and genotoxicity than their brominated and chlorinated counterparts, with iodoacetic acid identified as the most toxic. Trihalomethanes, which are disinfection by-products, present a variety of biological responses and mechanisms of toxicity in vivo and in vitro. The formation of adenomas, renal and liver adenocarcinomas, and hepatotoxicity are just a few examples of side effects. The accepted risk limit for potential toxic effects in drinking water is 10^−6^. In the case of kidney cancer development, this range is reached above 6 μg/L of bromodichloromethane concentration. Chlorinated and chloraminated drinking water from Canada and the USA showed iodotrihalomethane concentrations below the maximum threshold of 10.2 μg/L in most cases [36,49,50]. Exceeding the threshold concentration will be determined by the concentration of iodinated contrast media in the water sources employed for chlorination. Due to the extensive application of water disinfection methods, it is difficult to determine exact numbers. Although iodinated contrast agents exhibit low toxicity, their byproducts present during drinking water purification and in the environment can pose a risk to humans and the aquatic environment [51,52]. Assessing the toxicity of pharmaceutical metabolites requires consideration of their bioavailability. Metabolites may exhibit structural and polarity differences compared to their parent compounds, implying potential variations in their biological activity. Bioavailability is frequently linked to the K_ow_ (octanol–water partition coefficient), a parameter commonly used in QSAR analyses. QSAR methods have been applied to study physicochemical properties, providing insight into sorption behavior, aqueous solubility, volatility or environmental release of contaminants, which are associated with toxicity. However, based on the analysis of the obtained results, Differences between the experimental data and QSAR predictions were observed, therefore this approach is associated with certain limitations [53]. Effluent toxicity test (WETT), toxicity identification evaluation (TIE), and effect-oriented analysis using the bacterium *Aliivibrio fischeri* are also used to assess ecotoxicity [54]. Escher et al. (2005) [55] proposed additional toxicological testing toxicity tests using daphnia, algae, *Aliivibrio fischeri*, fish for six pharmaceuticals. These studies examined molecular interactions with flora and fauna, focusing on specific and nonspecific interactions related to endocrine disruption or photosynthesis inhibition, as well as reactive toxicity, including indirect reactions mediated by reactive oxygen species (ROS). Toxicity testing was performed on individual compounds, on mixtures of drugs with comparable mechanisms, and on random drug combinations to study their distinct effects. The results obtained enabled the determination of pharmaceutical toxicity and can be used to establish priorities in testing procedures. The ToxCast program, developed by the US Environmental Protection Agency (EPA), was used to assess the bioactivity profiles of environmental contaminants and their chemical properties using high-throughput assays and computational chemistry. This program can be used to prioritize pharmaceutical metabolites [55]. Another tool for assessing the ecotoxicity risks posed by pharmaceutical contaminants is literature data on pharmaceutical sales, the physicochemical characteristics of metabolites and parent compounds, along with the human metabolic and excretory pathways of drugs. Although some ecotoxicity data from QSARs for pharmaceuticals were estimated, information was not available. This model performed well in screening studies and showed that metabolites represent an important group of substances requiring assessment of their environmental risk. The suggested method validated that metabolites can pose an ecological risk [53]. It is important to note that advances in analytical instrumentation and recent advances in sample preparation, aimed at detecting previously unidentified contaminants with potential environmental hazards, accompany modelling approaches. For instance, fractionation techniques have been employed to isolate estrogenic compounds via bioassays. Other studies have described metabolic profiling approaches to detect endocrine-disrupting compounds and to assess bioactive metabolites. The outcomes of in vitro toxicity assays are influenced by both the combined concentrations of compounds and their pharmacological effects. Consequently, these factors are critical and should be taken into account during ecological and human health risk assessments. A similar approach to assessing pharmaceutical metabolites for endpoints (i.e., mutagenicity or chronic toxicity) besides estrogenic activity, it can be employed to detect environmentally relevant compounds. Continuous development and refinement of these tools, including metabolic profiling and bioactivity-based modelling techniques, could provide effective screening methods for identifying human metabolites and pharmaceuticals with high environmental risk [56]. Another issue is the potential impact of mixtures of pharmaceutical compounds from wastewater treatment plants on the host–parasite relationship in aquatic ecosystems.

Although iodinated contrast media generally exhibit low acute toxicity, increasing attention has been directed toward the potential risks posed by their metabolites and transformation products formed during drinking water purification and in the environment [51]. These by-products may differ substantially from their parent compounds in terms of structure, polarity, and reactivity, which can significantly influence their bioavailability and toxicological profiles. Bioavailability is commonly associated with the octanol–water partition coefficient (K_ow_), a key parameter used in quantitative structure–activity relationship (QSAR) analyses to estimate the potential biological activity and environmental behavior of contaminants. QSAR-based approaches have been applied to predict physicochemical properties such as sorption potential, aqueous solubility, volatility, and environmental distribution, which are indirectly linked to toxicity. However, discrepancies between experimental observations and QSAR predictions have been reported, indicating inherent limitations of modeling approaches when applied to complex pharmaceutical metabolites [57]. To complement predictive models, experimental ecotoxicological tools are increasingly employed to assess the toxicity of pharmaceutical metabolites and complex mixtures. Whole effluent toxicity tests (WETT), toxicity identification evaluation (TIE), and effect-oriented bioassays using organisms such as Aliivibrio fischeri are widely applied to evaluate the integrated toxic effects of treated wastewater and transformation products [11,58]. In addition, multi-species toxicity testing frameworks involving daphnids, algae, bacteria, and fish have been proposed to capture both specific and nonspecific modes of toxic action, including endocrine disruption, inhibition of photosynthesis, and reactive toxicity mediated by the generation of reactive oxygen species (ROS). Studies conducted on individual compounds, mixtures of pharmaceuticals with similar mechanisms of action, and randomly combined drug mixtures have demonstrated that transformation products and mixtures may exhibit additive or synergistic effects not predictable from parent compounds alone. High-throughput screening programs, such as the ToxCast initiative developed by the U.S. Environmental Protection Agency, provide further insights into the bioactivity profiles of environmental contaminants and their metabolites by combining in vitro assays with computational toxicology tools. These approaches allow for the prioritization of pharmaceutical metabolites based on their potential biological effects and exposure relevance [40,59]. Complementary screening strategies incorporating pharmaceutical consumption data, human metabolic pathways, and physicochemical characteristics of metabolites have also demonstrated that metabolites represent a critical and often underestimated group of environmentally relevant contaminants requiring risk assessment [41,47]. Recent advances in analytical chemistry and sample preparation techniques have further enhanced the identification of previously unknown bioactive metabolites. Effect-directed analysis, fractionation approaches, and metabolic profiling have been successfully applied to isolate endocrine-disrupting compounds and other biologically active transformation products in environmental samples. The outcomes of in vitro toxicity assays are strongly influenced by both the combined concentrations of compounds and their pharmacological interactions, emphasizing the importance of mixture toxicity considerations in environmental and human health risk assessments. Extending these approaches to additional toxicological endpoints, such as mutagenicity or chronic toxicity, may provide a more comprehensive evaluation of the environmental risks posed by iodinated contrast media metabolites [54,59,60]. Furthermore, the combined release of pharmaceutical mixtures from wastewater treatment plants may interfere with host–parasite relationships in aquatic ecosystems, highlighting the need for integrated ecological risk assessment frameworks.

Morley (2009) [57] collected toxicity data for parent pharmaceuticals, noting changes in host physiology and the risk of developing strains resistant to parasites following exposure to a mixture of medical and veterinary compounds. Based on the data presented, it can be assumed that if an avian influenza outbreak occurred, resulting in the intensive use of the Tamiflu vaccine, high concentrations of this drug in aquatic systems might contribute to the evolution of vaccine-resistant viruses in animals exposed to wastewater. Assessments of environmental risks from drugs and their metabolites have not considered interactions between hosts and parasites in aquatic organisms. However, for pharmaceuticals, numerous studies have been reported assessing the environmental risk to human health based on their hazard ratios, calculated as the ratio between predicted environmental concentrations (PECs) and predicted no-effect concentrations (PNECs). Although literature reports indicate that pharmaceutical levels in the environment are so low that they pose a negligible risk to humans, other researchers argue that antibiotics, in particular, can pose a significant risk, especially for the aquatic environment [47,61]. In summary, there is a lack of sufficient information regarding the chronic environmental impacts of pharmaceutical metabolites. This issue undoubtedly poses a challenge in refining models to improve their accuracy. Therefore, accurate risk assessment of pharmaceuticals and their metabolites necessitates analytical techniques sensitive to very low concentrations of environmental contaminants. Tackling iodinated contrast agents in water supplies depends on close cooperation between manufacturers and water providers. The first step is to create awareness among healthcare professionals and implement active collaboration. Radiologists, in particular, can help through the conscious and judicious use of contrast agents. There are several options. The first is the implementation of measures to reduce waste, optimize contrast agent use, and collect residual iodine at the point of use. The second approach is to decrease the environmental load of iodinated contrast agents by implementing techniques for processing and collecting urine in hospitals. More informed use of intravenous iodinated contrast agents can help mitigate this problem. During the 1980s and 1990s, an identical dose of contrast agent was administered to all patients, regardless of body weight [43]. Since then, numerous procedures have been introduced to determine the amount of contrast media necessary to achieve the intended imaging results. A growing number of institutions have implemented individual contrast volumes based on body weight and clinical history. Iodinated contrast agent prepared for administration to a patient but not used is discarded. This creates unnecessary costs and additional environmental burdens. For a large group of patients, injection systems allow the use of appropriately sized bottles covering the 50–500 mL range. This allows for individualized contrast agent administration without generating additional waste [62]. Another step is saline flushing. After contrast agent administration, the patient is injected with saline to “push” any unused contrast agent through the patient’s blood vessels to ensure that all of the contrast agent used contributes to the desired effect [52]. Separate collection of iodinated contrast agent residues and transferring them to specially designated hospital containers prevents these agents from entering the sewage system. These containers are then disposed of in an incinerator. This process contributes to the destruction of the contrast agent’s chemical composition and the generation of iodine salts and molecular iodine, which naturally occur in the environment. Furthermore, GE Healthcare offers an iodine recycling service. Custom containers are supplied to hospital radiology departments and subsequently retrieved by the company. The iodine is recovered and can subsequently be reused for the production of contrast agents. It is important to note that the contrast agent used is excreted in urine. To minimize the volume of waste discharged into the environment, urine can be collected in special bags, or hospital wastewater can be treated before being discharged into the sewer system. Single-use urine bags are equipped with an absorbent medium intended to retain urine. Patients employ them at home during the initial four voiding events after receiving intravenous contrast. The bags are thrown away with standard domestic garbage. If this type of waste is subjected to incineration, iodinated contrast media are reduced to iodine salts or molecular iodine. Iodinated contrast media that ends up in landfills are also unlikely to create an environmental hazard, provided they are properly managed. Additional urine collection after CT scans can be enabled through the installation of specially engineered toilets utilizing waterless urinal systems. These toilets need to be correctly connected to a dedicated storage tank. A sustainable strategy for managing pharmaceutical residues in hospital effluent is represented by the pharmafilter concept. This concept aims to treat liquid and solid hospital waste using a fermentation system within the hospital. This system operates by generating biogas and removing pharmaceuticals, including antibiotics. Studies conducted in the Netherlands have demonstrated the complete removal of iodinated contrast agents from hospital wastewater. However, implementing such a solution is technically feasible for newly built hospitals [63]. The limited availability of data on the long-term toxicity and chronic environmental effects of iodinated contrast media and their transformation products highlights the need for precautionary approaches. In this context, sensitization and awareness represent essential preliminary steps preceding the implementation of broader mitigation strategies, including recycling and advanced treatment technologies. Increasing awareness among healthcare professionals, manufacturers, and water management stakeholders is crucial to promote responsible contrast agent use and to support the adoption of sustainable practices. Educational initiatives, targeted training programs, and interdisciplinary communication can enhance understanding of the environmental pathways and potential risks associated with iodinated contrast agents, thereby fostering informed decision-making. Such awareness-driven actions provide the foundation upon which technical and organizational strategies for iodine waste reduction can be effectively developed and implemented [45,55,64]. It is important for radiologists to obtain the best possible image quality, which requires the use of a contrast agent. Despite this, many physicians lack knowledge regarding the effects of contrast agents on the aquatic environment. It is important to remember that healthcare workers also bear responsibility for using contrast agents. To increase awareness, it is essential that radiographers and radiologists participate in preventive programs to reduce the amount of contrast agents reaching water sources. Cooperation between all parties involved is essential. A collaborative approach, supported by the relevant authorities, is essential. The best solution would be for manufacturers, radiographers, radiologists, pharmacists, patients, and representatives of drinking water suppliers to collaborate on this issue. Sustainability is a growing concern in healthcare. Pharmaceutical manufacturers and hospitals aim to consistently minimize their environmental footprint and enhance sustainability practices. Pharmacists and physicians emphasize reducing drug waste and optimizing their use. Radiology offers significant potential for savings by implementing these initiatives. The worldwide demand for raw materials is steadily rising in parallel with the improving socioeconomic status of populations. Currently, most iodine required for contrast agent production is sourced from Japan and Chile, which contributes to the risk of production shortages and the exploitation of natural sources of iodine. Limited availability of raw materials has driven growing attention toward sustainable resource management [35,54]. The ability to recycle contrast agents from patient urine is certainly desirable. This contributes to resource savings and the creation of a closed loop. Bayer AG has introduced recycling processes to minimize the release of iopromide into wastewater treatment facilities. Recovering iodine in organic form helps reduce environmental or health risks to the aquatic environment and the demand for resources. The ever-expanding knowledge about the environmental impact of iodinated contrast agents and their degradation products contributes to understanding the biodegradation process and determining how to avoid toxic byproducts. Medical imaging techniques that require the use of contrast agents are constantly evolving. Most modern treatment methods, particularly in oncology, utilize contrast-enhanced computed tomography. In developed countries, improved access to imaging is contributing to the increased demand for contrast materials. Developing strategies to recover and reuse the raw materials used in manufacturing iodinated contrast media could become a significant challenge for the sustainable development of healthcare worldwide [52].

In recent years, increasing attention has been directed toward sustainable strategies aimed at reducing iodinated contrast media waste, reflecting the growing importance of sustainability across chemistry, biology, and medical technologies. Modern approaches prioritize source control and circular economy principles over end-of-pipe solutions alone. These strategies include optimized and individualized contrast dosing protocols, recovery of unused contrast agents at the point of care, and dedicated management of hospital effluents to limit the discharge of iodinated compounds into municipal wastewater systems. Additionally, the separate collection and treatment of contrast-containing urine, as well as decentralized on-site treatment concepts such as fermentation-based systems, have been proposed to significantly reduce iodine emissions from healthcare facilities. From a resource perspective, iodine recovery and recycling initiatives implemented by pharmaceutical manufacturers contribute to closing material loops, reducing reliance on limited natural iodine reserves, and minimizing the environmental footprint of contrast-enhanced imaging. Together, these measures demonstrate that sustainable iodine management requires integrated cooperation between healthcare providers, manufacturers, and water utilities, and represents a key pathway toward environmentally responsible diagnostic imaging practices [45,65,66].

Biological digesters have become a common and well-developed wastewater treatment method. These methods have been demonstrated to efficiently eliminate pharmaceuticals, including paracetamol, but they are not suitable for removing substances such as carbamazepine. To eliminate pharmaceuticals, it seems reasonable to combine biological processes with alternative treatment systems. Pharmaceutical compounds entering wastewater treatment plants are primarily removed during the biological treatment stage. The removal mechanism depends on parameters such as the biodegradability and hydrophobicity of the pharmaceuticals. Reports regarding these mechanisms vary: removal of contaminants through sorption processes, limited contribution of biological degradation, contribution of hydrolysis, and removal attributed to biotransformation. It should be noted that water entering wastewater treatment plants also contains metabolites derived from the metabolism of pharmaceuticals. These metabolites constitute a so-called reserve because, with the use of appropriate enzymes, they can be broken down into the parent pharmaceuticals [53]. Pharmaceutical compounds can be degraded through heterotrophic biodegradation, a process in which bacteria utilize organic carbon as a substrate, or by autotrophic biodegradation, in which bacteria convert NH_4_^+^ to NO_3_^−^ [66]. In the activated sludge process, both mechanisms occur concurrently, with the degradation pathway determined by the properties of the pharmaceuticals [55]. However, some researchers disagree with this issue regarding the relationship between pharmaceutical structure and biodegradability, pointing to the lack of correlation between compound structure and biological removal [67]. In the literature, the biodegradability of a compound is quantified using the biodegradation rate constant. Pharmaceutical sorption is generally faster than biodegradation, with equilibrium established in approximately one hour. Many authors highlight the significance of future research concentrating on the mechanisms of biodegradation and the interactions of pharmaceuticals to improve bioreactor efficiency. Biodegradation of pharmaceutical compounds can occur through substrate utilization or through co-metabolism. Examples include 17β-estradiol and ciprofloxacin, which biodegrade under these two conditions. Biological processing of pharmaceutical compounds can contribute to the degradation of shorter-chain products or mineralization, i.e., the transformation of contaminants into inorganic ions, water, carbon dioxide. Biotransformation and the formation of metabolites in pharmaceutical processing depend on specific conditions, including factors like the microbial species involved and the characteristics of the pharmaceuticals. The biotransformation process consists of lytic, reductive, and oxidative pathways. For instance, iopromide undergoes biotransformation in activated sludge treatment, leading to oxidation of the compound’s side-chain primary alcohols. Dehydroxylation of both side chains was observed in nitrifying activated sludge, connected to co-metabolic activity [68]. Identification of the biodegradation pathway relies on understanding the core metabolic processes, the transformation products formed, and the sequence in which they appear. Analysis of the products formed during iohexol biotransformation suggests that microbes drive decarboxylation reactions, N–C bond cleavage, and oxidation of primary and secondary alcohol sites. Enzymes dependent on thiamine pyrophosphate can promote decarboxylation. N–C bond scission occurs through the action of monooxygenases. Alcohol oxidation is induced by aldehyde and alcohol dehydrogenases [50,69,70]. The experimental assessment of biotransformation is a complicated procedure. Various methods are available to elucidate the structures and mechanisms of biotransformation pathways. One such example is the University of Minnesota’s Biocatalysis/Biodegradation database, which contains data on how transformation products of amide-containing pharmaceuticals are formed [71]. An additional example was described by [49], who presented the identification of transformation products with anticancer and antimitotic activity. In addition to physicochemical treatment technologies, biological processing represents a key component of contemporary strategies for the removal of pharmaceutical contaminants from wastewater. Biological digesters and activated sludge systems are widely applied due to their cost-effectiveness and environmental compatibility, and they have demonstrated high removal efficiencies for readily biodegradable pharmaceuticals. However, the effectiveness of biological treatment strongly depends on the molecular structure, functional groups, and bioavailability of the compounds. While substances such as paracetamol are efficiently degraded, more persistent pharmaceuticals, including carbamazepine and many iodinated contrast media, exhibit limited removal. To address these limitations, hybrid treatment concepts combining biological processes with complementary technologies have been proposed. Biological removal mechanisms include heterotrophic biodegradation, autotrophic nitrification-linked processes, co-metabolism, and enzymatic biotransformation, occurring simultaneously within activated sludge systems. These processes may lead to partial degradation, formation of transformation products, or complete mineralization to inorganic end products such as CO_2_, H_2_O, and inorganic ions. Recent studies emphasize that microbial community composition, redox conditions, and enzymatic activity play a decisive role in determining biodegradation pathways and efficiencies. Consequently, optimizing bioreactor design, hydraulic retention times, and microbial adaptation is considered a promising direction for enhancing the biological processing of pharmaceutical contaminants, including iodinated contrast agents, in wastewater treatment plants [56,72,73]. Ultrasonic processing represents an emerging treatment technology for the removal of pharmaceutical contaminants from water and wastewater systems. The application of high-frequency ultrasound induces acoustic cavitation, leading to the formation, growth, and collapse of microbubbles, which generate localized extreme conditions of high temperature and pressure. These conditions promote the formation of reactive species, such as hydroxyl radicals, enabling the degradation of pharmaceutical compounds through sonochemical oxidation. Ultrasonication has been shown to effectively degrade a range of pharmaceuticals, particularly when combined with other advanced treatment processes; however, its standalone application is often limited by high energy demand and incomplete mineralization, resulting in the formation of transformation products. Membrane-based technologies constitute another important class of advanced treatment methods used for pharmaceutical removal. Processes such as nanofiltration (NF), reverse osmosis (RO), and, to a lesser extent, ultrafiltration (UF) enable the physical separation of pharmaceutical compounds based on size exclusion, charge interactions, and hydrophobicity. Membrane systems are highly effective in removing a broad spectrum of pharmaceuticals, including persistent and poorly biodegradable compounds, and are increasingly applied in drinking water treatment and water reuse schemes. Nevertheless, membrane fouling, concentrate management, and operational costs remain key challenges limiting large-scale implementation. Hybrid systems combining membrane filtration with biological treatment, ultrasonication, or advanced oxidation processes have therefore been proposed to enhance overall removal efficiency while mitigating individual process limitations. These integrated approaches highlight the growing role of ultrasonication and membrane-based technologies within multi-barrier strategies for controlling pharmaceutical contamination in aquatic environments [74,75,76,77]. This method is used to remove pharmaceuticals from hospital and municipal wastewater. The degradative effect is related to the phenomenon of acoustic cavitation. This phenomenon involves the rapid collapse of bubbles. Elevated pressure and temperature develop within the bubble, leading to water molecule dissociation and the formation of reactive radicals that aid in the breakdown of organic compounds. Such breakdown can occur within the bulk solution, at the interface between bubble and solution, and inside the bubble itself. The extent of degradation is influenced by the compound’s hydrophilic, hydrophobic, and volatile properties, all of which are affected by the solution conditions. Acoustic cavitation used to eliminate pharmaceutical compounds can be induced by horn-type sonicators and disk transducers. These laboratory-scale reactors are operated with treatment volumes of up to one litre. The extent of degradation is influenced by sonication factors—including calorimetric power density, frequency, irradiation mode, and reactor configuration—along with solution parameters such as temperature, pH, concentration, and the characteristics of the contaminant. Membrane filtration processes represent another approach capable of effectively removing particulate matter at elevated concentrations. Biological treatment approaches, such as membrane bioreactors, have been effectively enhanced through the incorporation of microfiltration and ultrafiltration membranes. Nevertheless, these membranes do not offer a complete barrier to pharmaceutical compounds. Techniques such as osmosis, reverse osmosis, and nanofiltration can effectively eliminate pharmaceuticals, yet highly concentrated solutions require further processing. The choice of filtration type is determined by the properties of the contaminant, including its charge, molecular size, and hydrophilic or hydrophobic nature. Available literature data confirm that biological purification methods are not highly effective in removing compounds such as carbamazepine due to their limited interaction with suspended solids in mixed liquids. Ultrasound is effective in degrading pharmaceutical compounds, but for some of them, sonication alone is not sufficient for complete mineralization. Sonication results in the production of intermediates that are markedly more toxic than the original compounds. However, the obtained intermediates can lower resistance to antimicrobials and increase biodegradability. Osmosis, reverse osmosis, and nanofiltration have demonstrated significant separation of pharmaceuticals, but they do not constitute a complete barrier. It is hypothesized that optimization of these methods can be achieved by combining filtration systems with ultrasound and bioreactors or through the integration of ultrasound with advanced oxidation methods [70,72].

Iodinated contrast media are continuously introduced into aquatic environments as a consequence of their widespread medical use and insufficient removal during conventional wastewater treatment. Their high water solubility and chemical stability result in pseudo-persistent behavior in surface waters, groundwater, and drinking water resources. Although parent ICM generally exhibit low acute toxicity, their continuous release, chronic exposure, and occurrence in complex mixtures raise environmental concerns. Hospital effluents and wastewater treatment plant discharges represent the primary pathways of environmental dissemination, emphasizing the growing relevance of ICM as emerging aquatic contaminants and the need for long-term monitoring and improved management strategies.

## 3. Chemical Fate of ICMs

### Transformations of Commonly Used Iodine Contrast Media

Iodinated contrast agents get into drinking water because standard wastewater treatment technologies fail to adequately eliminate them. Even low concentrations of iodine my promote the formation of disinfection by–products (DBPs). The most commonly detected by-products are the compounds chloroform, dichloroacetic acid, trichloroacetic acid.

**Figure 3:**
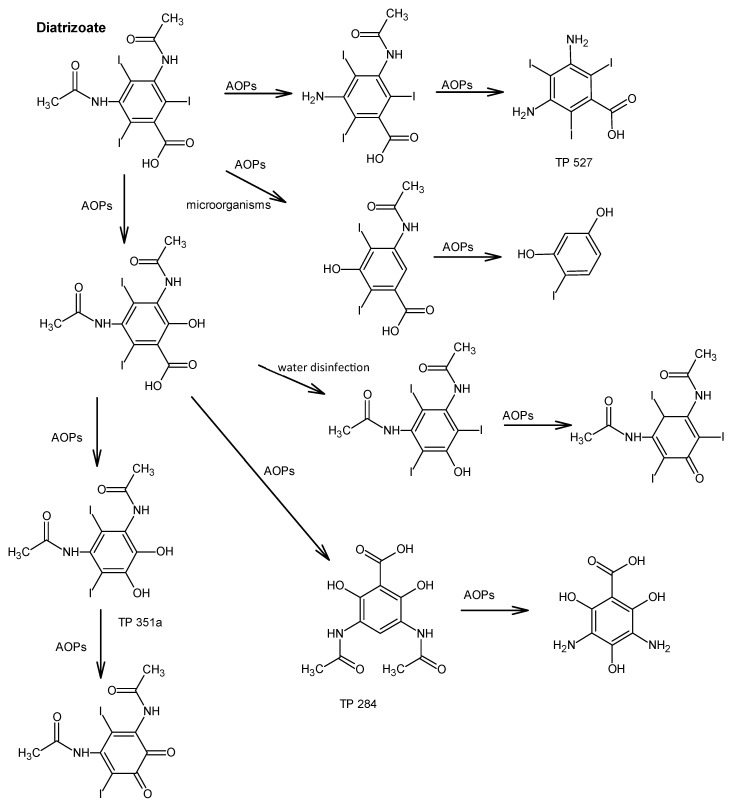
Proposed transformation pathways for diatrizoate subjected to advanced oxidation processes (AOPs) [11,56,63,64,65,66].

**Figure 4:**
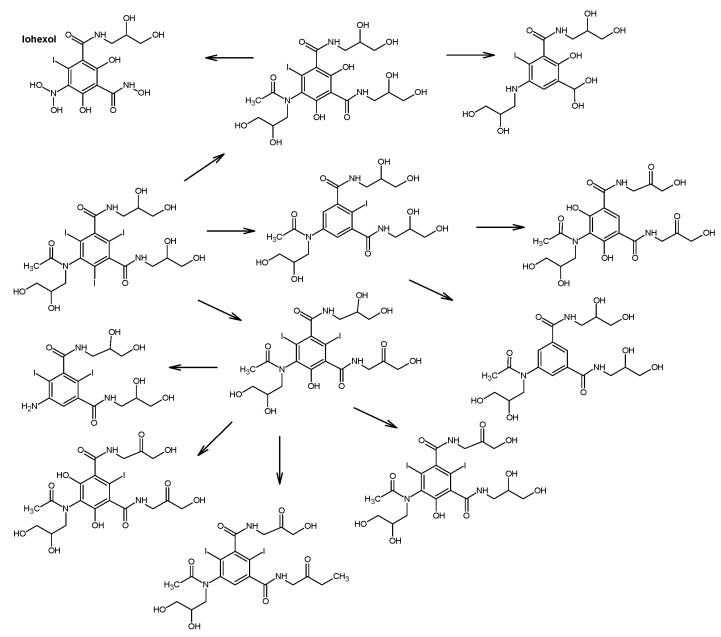
Proposed transformation pathways for iohexol subjected to advanced oxidation processes (AOPs) [11,56,59,63,64,65,66].

**Figure 5:**
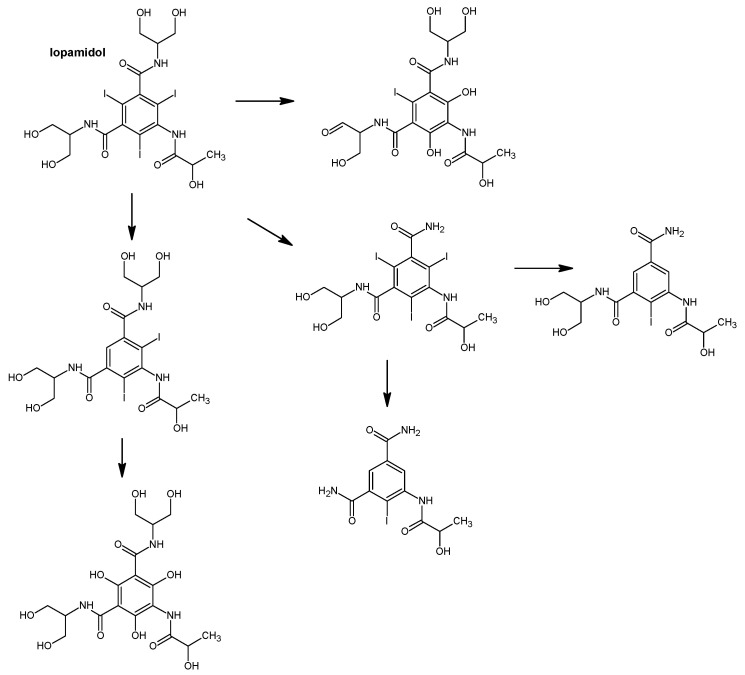
Proposed transformation pathways for iopamidol subjected to advanced oxidation processes (AOPs) [11,56,59,63,64,65,66].

In addition, various iodinated disinfection by–products (I–DBPs) may also generated. One of these is iodo–trihalomethanes (I–THMs). These compounds can result in undesirable taste and odor in potable water. In the first phase of raw water disinfection, iodine is converted to hypoiodous acid (HOI) when reacting with chloramine or chlorine. HOI interacts with organic compounds to form CH_3_I, among others [43]. Water parameters, i.e., temperature, pH, organic matter concentration or iodine concentration, impact the generation of CHI_3_ in the course of water treatment processes. The production of I-THMs in water treated with chloramine or chlorine in the presence of iodinated contrast agents (iohexol, diatrizoate, iopromide) was dictated by the availability and concentration of monochloramine and chlorine. After chloramination with the addition of NH_2_Cl to water containing iopamidol, six different iodo–trihalomethanes were identified—CHI_3_, CHBrI_2_, CHBr_2_I, CHBrI, CHClI_2_, CHCl_2_I. In contrast, during chlorination using Cl_2_, only three I–THM—CHBrClI, CHCl_2_I, CHCl_2_—were identified [78]. Figure 3, Figure 4 and Figure 5 show the proposed degradation pathways of the most commonly used contrast agents.

The chemical fate of iodinated contrast media is largely governed by their high stability and resistance to hydrolysis, photolysis, and biodegradation under environmentally relevant conditions, which limits their removal during conventional biological wastewater treatment. While advanced oxidation processes can effectively degrade parent ICM, they often lead to the formation of transformation products rather than complete mineralization. Of particular concern is the formation of iodinated disinfection by-products during drinking water treatment, which may exhibit higher toxicity than the parent compounds. Consequently, environmental risk assessment of iodinated contrast media should not be restricted to the parent substances alone but must also account for transformation products formed under different redox and treatment conditions.

## 4. Advanced Oxidation Processes (AOPs)

**Figure 6:**
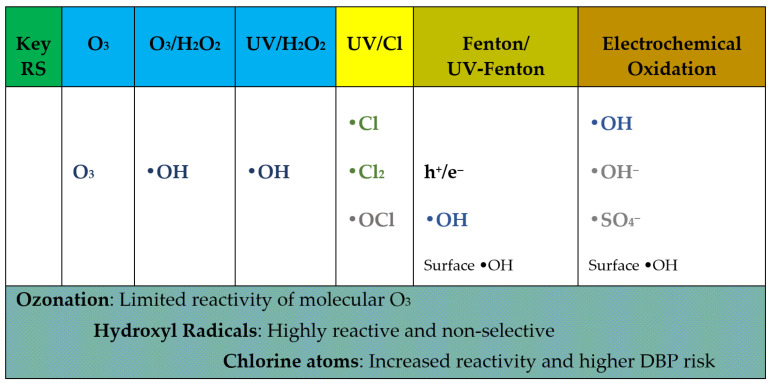
Schematic summary of dominant reactive species (RS) in Advanced Oxidation Processes for ICM Degradation.

The use of advanced oxidation processes is regarded as one of the most effective strategies for removing iodinated contrast agents from hospital wastewater. An AOP uses sulfate (SO_4_·^−^) and hydroxyl (-OH) radicals to break down persistent organic compounds. Hydroxyl radicals are characterized by the fact that they are highly reactive, reacting with contaminants with kinetic constants of 10^6^–10^9^ L mol^−1^s^−1^ [78]. The degradation of iodine contrast agents has been studied very extensively in recent years. In laboratory studies, ozonation, photocatalysis with TiO_2_, UV photolysis, UV/H_2_O_2_ have been proven to be highly effective in degrading compounds such as iopamidol, iohexol, and iopromide. Advanced oxidation methods are based on UV radiation and ozone, together with physical, catalytic processes and electrochemical [78,79,80,81,82,83,84,85,86,87]. The selected examples based on recent literature data are enlisted in Table 3. Schematic overview of the dominant reactive species (RS), including OH, SO_4_^−^, and reactive chlorine species, responsible for iodinated contrast media (ICMs) degradation in selected advanced oxidation processes is depicted in Figure 6.

### 4.1. Ozonation (O_3_)

Advanced ozone-based oxidation processes lead to the generation of hydroxyl radicals. They can be divided into ozonation, O_3_/H_2_O_2_ peroxide process and O_3_/UV combination. Ozone, because it can produce hydroxyl radicals and features involving molecular oxidation, has a wide range of performance in water disinfection and micropollutant removal. However, studies on the degradation of iodine contrast agents using ozone showed high resistance of these compounds to ozonation. It was found that O_3_ does not oxidize iodine contrast agents, while due to the formation of hydroxyl radicals, 50% degradation efficiency can be attained at an O_3_ level of 5 mgL^−1^. However, even at such a high concentration, degradation of diatrizoate, which appears to be less reactive to hydroxyl radicals, was not achieved [71]. Seitz et al. (2008) [86] used various liquid chromatography methods in their study to characterize by–products after the ozonation process. Compounds containing carbonyl and aldehyde groups were recognized as stable by–products during iomeprol transformation. Because iodine contrast agents show partial reactivity to ozonation, it has been shown that by adding a catalyst or reactant, the level of mineralization during ozonation can be increased. Yan et al. (2021) [87] implemented a catalytic ozonation process to improve iohexol elimination. Both goethite and a goethite–magnetite composite material demonstrated potential as catalysts. This resulted in a 16.5% increase in the removal rate for goethite, and a 21.2% increase for the composite compared to salt ozonation. Combining ozone with hydrogen peroxide aims to improve the elimination of iodine-based contrast agents. It relies on hydrogen peroxide’s ability to form hydroxyl radicals compared to ozone alone. The degradation of iopamidol, iopromide, iomeprol and diatrizoate using ozone and ozone with hydrogen peroxide was studied under laboratory conditions. During ozonation, enhancement of the radical mechanism was noted, By adding hydrogen peroxide, 80% of diatrizoate was effectively removed and complete removal of non-ionic compounds, with a low oxidant mass ratio [88,89,90]. In addition, ozone-based radical oxidation contributed to the release of inorganic iodine. The lower ozone-induced reactivity of diatrizoate was confirmed in other studies, in which ionic diatrizoate showed a removal efficiency of about 24%. Non-ionic contrast compounds were removed in more than 80% with 10 mgL^−1^ ozone. Due to the low removal efficiency of iodine contrast agents under O_3_ alone and with H_2_O_2_/O_3_ combination, other advanced oxidation processes were analyzed to obtain much higher removal efficiencies. Diatrizoate removal was also studied by an electrochemical advanced oxidation process that combines ozonation with cathodic production of hydrogen peroxide. Catalyst-supported diffusion cathodes were constructed from modified carbon nanotubes. A significant degradation efficiency of 70% was thus achieved [75,76,91,92,93]. The ozonation process can lead to partial degradation of iodinated contrast agents; however, many literature reports indicate that ozone alone is not effective enough. Ozone reacts with unsaturated sites, which are rarely present in the structure of these compounds. Rühmland et al. (2015) [56] using typical ozone doses, achieved about 30% degradation of iopromide in their study. Certainly the immeasurable advantage of ozonation is the speed of the reaction, but due to the high cost and the possibility of by-products, it is recommended to combine this method with other techniques.

### 4.2. UV/H_2_O_2_

Removal of ionic contrast agents during UV/H_2_O_2_ treatment occurs through oxidation by non-selective hydroxyl radicals generated in situ and by direct photolysis under 254 nm UV light. Degradation of iodine contrast agents was investigated using UV-based photolysis processes. Photolysis at a UV dose of 160 mJ·cm^−2^ resulted in about 40% degradation of 0.5 μmol·L^−1^ diatrizoate. In contrast, the efficiency of diatrizoate degradation indicated a slight acceleration with the addition of H_2_O_2_ (excess H_2_O_2_ would compete with the degradation of iodine contrast agents and consume hydroxyl radicals). However, improved performance was observed with other contrast agents like iohexol. While UV/H_2_O_2_ treatment did not achieve substantial mineralization of iopromide, the resulting by-products underwent biodegradation. This suggests that coupling with biological treatment is feasible for this compound [11,94].

### 4.3. UV/Cl

The UV/Cl process is regarded as a promising substitute for the UV/H_2_O_2_ method due to the production of radicals, including •Cl, •Cl_2_, •OCl, and •OH, which are generated during the photolytic breakdown of hypochlorous acid (HOCl) and hypochlorite ions (OCl^−^). Several studies have further investigated the degradation kinetics of iodine contrast agents including iopromide, iopamidol, iohexol and diatrizoate. Despite the presence of chlorine, diatrizoate was not significantly degraded, 90% removal efficiency was achieved using UV light at 10 min and the compound was completely removed after 3 min using the UV/chlorine process. Significant diatriazate removal efficiency has been documented in previous studies. Nevertheless, the generation of carcinogenic compounds, including chloroacetonitrile, has been reported, highlighting the need for additional treatment to mitigate the toxicity of the by-products [95].

### 4.4. Photocatalysis (UV/TiO_2_)

Mineralization of iodine contrast agents such as iopromide, iopamidol, iomeprol, diatrizoate was studied using photocatalysis. Photocatalysts including TiO_2_, Fe_2_O_3_, ZnS, CdS, ZnO, were used due to their low water solubility, high chemical stability and low cost. Titanium dioxide (TiO_2_) is the most widely employed photocatalyst. The mechanism of degradation of organic pollutants using photocatalysis is well known. Ultraviolet light with an energy exceeding the energy of the bandgap of the TiO_2_ semiconductor is used for this process. Borowska et al. (2015) [79] also studied iohexol in water. They obtained the best results after a time of 60 min and at pH 5–6. The study additionally monitored degradation by-products using LC-HRMS/MS. The analysis included phenols and carboxylic acids. A rise in toxicity levels was observed, which may indicate the formation of intermediate metabolites that are more biologically active than the starting compound [80,85].

### 4.5. Fenton Processes

The Fenton process, discovered by Henry J. Fenton, involves the activation of hydrogen peroxide with iron salts to facilitate the oxidation of tartaric acid. This process has been widely studied in the context of wastewater treatment, primarily because of its low cost. The oxidation degradation of organic compounds which act as pollutants, gives rise to intermediate species that can be oxidized to inorganic salts, CO_2_ and H_2_O [92,93,96]. Literature reports indicate that diatrizoate oxidation with Fenton’s reagent was performed at a temperature of 20 °C, varying the initial concentrations of hydrogen oxide and ferric ions. Removal of diatrizoate at a concentration of 3 mgL^−1^ obtained after 5 min was 41.5% using 0.5 mmol L^−1^ Fe^2+^ and 0.5 mol L^−1^ H_2_O_2_. By varying the conditions of 1.5 mmol L^−1^ H_2_O_2_ and 0.1 mmol L^−1^ Fe^2+^, a higher removal percentage of 72% was obtained. Literature reports indicate that diatrizoate removal efficiency is more strongly affected by the initial H_2_O_2_ concentration than by Fe^2+^ levels [82]. Additionally, FeO has been investigated as a catalyst for activating H_2_O_2_ in the removal of iopamidol. Complete removal of 2 mg L^−1^ iopamidol was achieved using zero-valent iron in the presence of 1 mmol L^−1^ H_2_O_2_. In contrast, the degradation efficiency dropped to merely 9.5% when FeO was not present. While the Fenton process has been the subject of considerable research and has been found to be effective in the purification of contaminants exuded by iodine contrast agents, it has some identified drawbacks, such as the difficulty in recycling a homogeneous Fe^2+^ catalyst, the significant volume of iron precipitate produced and the limited optimal pH range. Therefore, to achieve the production of hydroxyl radicals from hydrogen peroxide, research should be conducted to find economically viable and practically acceptable Fenton catalysts [97,98]. Elmolla & Chaudhuri (2010) [98] conducted a study on the degradation of iomeprol over 60 min, obtaining more than 80% degradation of this compound. Kormos et al. (2010) [48] used UV/Fenton and UV/H_2_O_2_ in degradation studies of iohexol, obtaining degradation rates of around 80%. In addition, deiodinated products and aromatic compounds were detected and monitored by LC-MS/MS.

### 4.6. Electrochemical Processes

Electrochemical techniques constitute a separate class of advanced oxidation methods, wherein reactions are initiated by the application of an electric current that flows between an electrode system and a solution or through the area between an electrochemically controlled working electrode. The degradation process proceeds through direct contact between the target compound and the electrode surface, resulting in the consumption or release of electrons. Another mechanism may contribute reductants or oxidants that react with the target impurity, but the reaction does not necessarily take place at the electrode–solution interface. In anodic oxidation, degradation can proceed via heterogeneous reactive oxygen species produced from water oxidation, direct electron transfer at the electrode surface, and the action of oxidants such as O_3_ or H_2_O_2_. The performance of anodic oxidation is largely determined by the anode material. Over the last twenty years, numerous anode materials have been investigated for the degradation of iodinated contrast agents. Broadly, anodes are categorized as either inactive, with high oxygen overpotential, or active, with low oxygen overpotential. The elevated oxygen evolution potential of inactive anodes reduces hydroxyl radical interactions with the electrode surface, thereby enhancing their chemical reactivity toward organic pollutants [99]. Zhang et al. (2013) [100] conducted a study of electrochemical reduction of diatrizoate. The study used graphite felt and felt with palladium nanoparticles. The degradation efficiency significantly increased in the presence of palladium nanoparticles, achieving complete deiodination. In addition, electrochemical oxidation using boron-doped diamond anodes contributed to highly efficient by-product mineralization. However, this process requires careful optimization on real-world samples in the future. In a study by Rizzo et al. (2013) [101], boron-diamond anodes were used, and about 90% degradation of iopromide was achieved in 30 min, with appropriately selected voltages. However, the disadvantages of this method are high cost and the formation of by-products.

### 4.7. Synthetic Remarks on AOPs for ICMs Treatment

**Figure 7:**
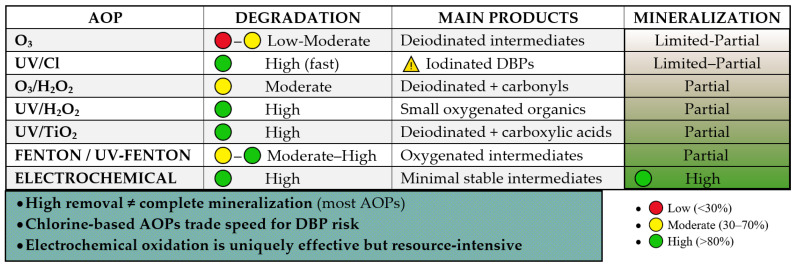
Comparative overview of degradation efficiency and major transformation products formed during treatment of iodinated contrast media by different advanced oxidation processes.

While individual advanced oxidation processes (AOPs) have demonstrated varying degrees of effectiveness in degrading iodinated contrast media (ICMs), a comparative assessment reveals substantial differences in their ability to achieve mineralization, control toxic by-product formation, and meet practical implementation requirements (Figure 7).

**Figure 8:**
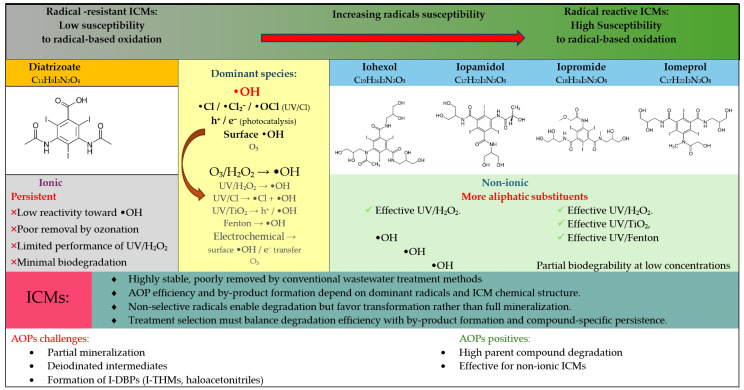
A visual summary of reactivity of ICM towards radical based oxidation (×-negative effect, ✓-positive effect).

Across most AOPs, degradation of parent ICM molecules is primarily driven by non-selective hydroxyl radicals (∙OH), resulting in deiodination and partial oxidation rather than complete mineralization. The susceptibility of featured ICMs towards decomposition by reactive species is visually summarized in Figure 8. The inherently high iodine content of ICMs promotes oxidative deiodination, leading to the release of inorganic iodine, which may subsequently participate in halogenation reactions, particularly during downstream chlorination or chloramination. Ozonation alone is generally ineffective for highly iodinated and aromatic ICMs such as diatrizoate, owing to the absence of ozone-reactive unsaturated bonds in their molecular structure. Although the addition of H_2_O_2_ or suitable catalysts enhances hydroxyl radical generation and improves parent-compound degradation, mineralization remains limited and stable oxygenated intermediates—such as aldehydes and carboxylic acids—often persist. Moreover, iodide released during ozone-based treatment increases the risk of forming iodinated disinfection by-products (I-DBPs) in subsequent disinfection steps. Among these, iodo-trihalomethanes such as iodoform (CHI_3_) have been reported and are associated with taste and odor problems as well as elevated cytotoxicity compared to their chlorinated analogues. As shown in Figure 8, ICMs generally exhibit high reactivity towards hydroxyl radicals (OH), while their reactivity towards sulfate radicals (SO_4_∙^−^) and other selective radical species is more compound-specific and structure-dependent. The presence of electron-withdrawing iodine substituents and stable amide functionalities significantly influences the susceptibility of ICM molecules to radical attack.

**Figure 9:**
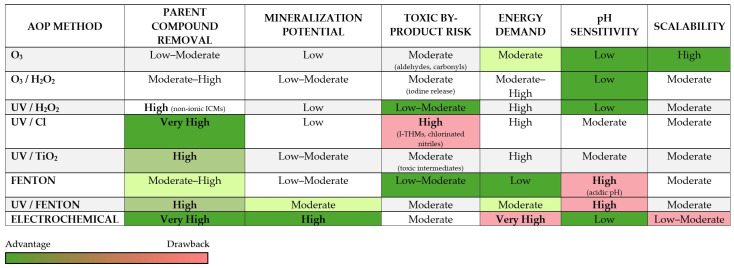
Comparison of selected processes for ICM removal.

UV-based processes, including UV/H_2_O_2_ and UV/TiO_2_, provide higher degradation efficiencies for non-ionic ICMs such as iopamidol, iohexol, and iopromide. These systems predominantly rely on hydroxyl radical chemistry and generally produce fewer halogenated by-products during the oxidation step itself. However, treatment typically results in partial oxidation, yielding deiodinated aromatics, phenols, and carboxylic acids. Several of these intermediates have been shown to exhibit increased biological activity or toxicity relative to the parent compounds and may act as precursors for iodinated disinfection by-products during subsequent chlorination, highlighting that high removal efficiency does not necessarily equate to reduced environmental risk. In contrast, the UV/Cl process achieves rapid and, in some cases, near-complete degradation of ICMs, including compounds that are otherwise resistant to oxidation. This enhanced performance is offset by a pronounced risk of forming toxic halogenated by-products due to the involvement of chlorine-centered radicals (Cl, Cl_2_^−^,∙OCl). Numerous studies have reported the formation of chlorinated nitriles, such as chloroacetonitrile, as well as iodinated and mixed iodinated–chlorinated species, including iodo-trihalomethanes (e.g., CHI_3_). These compounds are associated with cytotoxicity, genotoxicity, and carcinogenic potential, necessitating additional downstream treatment or polishing steps to mitigate by-product-related risks. Fenton and UV/Fenton processes offer relatively high degradation rates at comparatively low operational cost and are effective for a range of non-ionic ICMs. However, their practical application is constrained by a narrow optimal pH range, the generation of iron-containing sludge, and limited mineralization. While these processes are generally less prone to forming halogenated disinfection by-products, they frequently yield partially oxidized, deiodinated aromatic intermediates, and toxicity assays have, in some cases, indicated a temporary increase in ecotoxicological effects during treatment. Among the reviewed methods, electrochemical oxidation—particularly using boron-doped diamond anodes—shows the greatest potential for deep oxidation and near-complete mineralization of iodinated contrast agents, thereby minimizing the accumulation of persistent and toxic intermediates. Nevertheless, high energy demand, electrode material costs, and operational complexity currently limit large-scale implementation. Furthermore, under suboptimal operating conditions, the release of inorganic iodine and the transient formation of halogenated intermediates cannot be entirely excluded [102,103]. Overall, this comparison demonstrates that no single advanced oxidation process simultaneously maximizes mineralization, minimizes toxic by-product formation, and satisfies practical and economic constraints. Processes involving chlorine or producing free iodine pose a particularly high risk of generating toxic halogenated by-products, whereas hydroxyl radical–based systems without halogen involvement generally present lower DBP risks but may still yield biologically active intermediates. Consequently, hybrid treatment trains-combining AOPs with biological treatment, adsorption, or membrane processes, represent the most promising strategy for the sustainable and effective removal of iodinated contrast agents from complex wastewater matrices. A comparative evaluation of selected AOPs for ICM removal is presented in Figure 9; however, this assessment is primarily based on laboratory- and pilot-scale studies. The evaluation of toxic by-product risk includes iodinated and chlorinated disinfection by-products, such as iodo-trihalomethanes and chlorinated nitriles, as well as biologically active intermediate transformation products. It must be added that the conventional treatment processes, described in the next chapter, such as biological treatment or adsorption generally exhibit limited efficiency towards ICM removal, primarily due to the high polarity and chemical stability of these compounds. In this perspective, advanced oxidation processes demonstrate higher removal efficiencies, although their performance strongly depends on process configuration and operational conditions.

## 5. Conventional Methods

Iodinated contrast agents are extremely resistant to standard wastewater treatment processes. Available literature studies show that in municipal wastewater treatment plants (WWTPs), ICM removal does not exceed 20% [28,44]. The most common method at the secondary and tertiary treatment level is adsorption on activated carbon. In a study by [103], activated carbon and dusty carbon were shown to be moderately effective in removing ICM, influenced mainly by chemical structure and to a lesser extent by environmental conditions. Iopromide and iomeprol will be more susceptible to adsorption than diatrizoate. Due to the environmental permeation of iodine contrast agents and their considerable persistence after conventional wastewater treatment, posing challenges for the degradation of concentrated effluents (hospital waste, internal wastewater and industrial wastewater), would improve pollution control. High-concentration removal of iodine contrast agents can be achieved by prior concentration using a membrane process or absorption. Necessary properties to select the optimal adsorbent include uniform pore structure combined with elevated specific surface area, chemical inertness, substantial pore volume, thermal stability and controlled structure. Alumina, silica, activated carbon, zeolite and composite materials have exhibited significant adsorption performance in the removal of contaminants. Widely available and inexpensive adsorbents, i.e., spent tea leaves, marine microalgae, agro-industrial waste, also show promise for contaminant cleanup [65,104,105]. Despite this, few studies have addressed the adsorption of iodinated contrast agents. Therefore, The adsorptive removal of diatrizoic acid was studied using superparamagnetic iron oxide nanoparticles functionalized with methacrylic acid, SiO_2_ and Al(OH)_3_. The study reported a maximum removal efficiency of approximately 60% when 0.05 g of adsorbent was applied to 0.5 L of influent at pH 8 after 7 h of incubation. Membrane separation provides an alternative strategy for concentrating organic compounds. In their investigation of pharmaceutical removal from hospital wastewater, highlighted the importance of concentrating micropollutants through nanofiltration to enhance their degradation by electrochemical oxidation [100]. Among the existing methods that have been tested to treat iodine contrast agent–containing wastewater, biodegradation is considered both economically viable and environmentally friendly. Aerobic microorganisms have the ability to degrade specific organic pollutants. They grow rapidly and can mineralize organic compounds, using them as both energy and carbon sources. The literature describes studies of low concentration biotransformation of iodine contrast agents. Iopromide at concentrations ranging from 0.10 to 0.27 μg L^−1^ can be effectively removed via biotransformation. In activated sludge containing nitrifying bacteria, removal efficiency reached 97%, accompanied by the formation of dehydroxylated iopromide as a metabolite. Inhibition of nitrifying bacteria reduced the removal efficiency to 86%, producing a carboxylate derivative as the primary metabolite. After 54 h of incubation in fresh activated sludge, about 85% of iopromide—1.85 nmol L^−1^ was biotransformed, although the metabolites were not identified. In contrast, degradation of diatrizoic acid under the same conditions was minimal [98]. Tests on the biodegradability of diatriozic acid were also conducted using biological simulation of wastewater treatment and the Zahn—Wellens test. The biological simulation did not yield the expected results, while the Zahn—Wellens test biotransformed diatriose acid to 2,4,6-triiodo-3,5-diamino-benzoic acid [106]. Kormos et al. (2010) [48] studied biotransformation in aerobic soil using typical iodine contrast agents (iopamidol, iomeprol, iohexol, diatriose acid). After a process lasting almost six months, biotransformations of iopamidol, iomeprol and yohexol were noticed at neutral pH, only diatriose acid was persistent. Biotransformation was carried out in a wetland pond with floating plants, where bioaccumulation of diatriozic acid was also observed [107]. The combination of membrane separation and activated sludge processes was also carried out developed at Rensselaer Polytechnic Institute and brought to market in the late 1970s and early 1980s. The membrane modules and bioreactor serve two important functions. The initial method involves the microbial biodegradation of organic pollutants conducted in a bioreactor. The second function is the separation by means of a membrane module of microorganisms from the wastewater following treatment. The membrane bioreactor was used for a year in a Swiss hospital to be able to study and determine the elimination efficiency of iodine contrast agent micropollutants. The maximum elimination efficiency was recorded for iopromide (31%), while the other compounds exhibited minimal or negligible removal [107]. Although biodegradation studies of organic pollutants, i.e., personal care products or pharmaceuticals, were conducted under aerobic conditions, anaerobic conditions may also prove interesting for diatriozic acid bioaccumulation. Redeker et al. (2014) [107] conducted batch experiments with soil and sludge in which they found that under anaerobic conditions diatriozic acid can be removed, a process that leads to the formation of seven by-products. Learning about the degradation pathways, proved that in addition to deiodination, deacetylation also occurs. Nevertheless, 3,5-diaminobenzoic acid, identified as a by-product, exhibited stability under anaerobic conditions. The anaerobic transformation of iopromide was further studied in batch water–sediment systems under an argon atmosphere within a glove box. Iopromide (2.6 μmol L^−1^), after 20 days of incubation, nearly completely removed and underwent dehalogenation catalyzed by reductive deiodinase. In addition, the anilide groups were hydrolyzed. The main transformation of iopromide was sequential deiodination, resulting in the generation of five transformation by-products. The removal efficiency of diatriozic acid was negligible under conventional conditions. However, with iopromide, significant improvements can be achieved under conditions such as activated sludge containing bacteria that oxidize ammonia. Using aerobic biological treatment, high concentrations of iodine contrast agents do not appear to be effective, due to the fact that only selected iodine contrast agents can be biotransformed at low concentrations. Anaerobic purification appears to be more effective because dehalogenation can occur. Nonetheless, it should be emphasized that such investigations have been limited to low concentrations of iodinated contrast agents. Biological treatment cannot lead to complete mineralization, so studies have been conducted on physicochemical processes [58].

## 6. Conclusions

Iodinated contrast agents are widely employed in medical imaging and are increasingly found in aquatic environments owing to their environmental persistence and inadequate removal by conventional wastewater treatment processes. Although ICMs themselves show low acute toxicity, their transformation during water disinfection can lead to the formation of iodinated disinfection by-products with cytotoxic and genotoxic properties.

Advanced oxidation processes have emerged as the most promising methods for ICM degradation and mitigation of I-DBP formation. These processes, based on the generation of highly reactive species, effectively break down stable organic molecules. Techniques such as ozonation, UV photolysis, UV/H_2_O_2_, and photocatalysis with TiO_2_ have demonstrated high efficiency in degrading ICMs like iopamidol, iohexol, and iopromide in laboratory studies.

Despite their potential, further optimization of AOPs is required to enhance degradation efficiency under real wastewater conditions, minimize energy consumption, and prevent the formation of toxic intermediates. Integrating AOPs into existing treatment systems represents a key step toward sustainable and effective removal of iodinated contrast agents from hospital and municipal effluents, contributing to improved water quality and reduced environmental impact.

## Figures and Tables

**Table 1:** Characteristics of widely used iodinated contrast agents.

	Molecular Formula	Weight, g/mol	Charge	Solubility, mg/mL	Melting Point, °C	Osmolality
Diatrizoate	C_11_H_9_I_3_N_2_O_4_	613.916	Ionic	0.107	261–262	High
Iohexol	C_19_H_26_I_3_N_3_O_9_	821.142	Non-ionic	0.796	174–180	Low
Iopamidol	C_17_H_22_I_3_N_3_O_8_	777.089	Non-ionic	0.117	300	Low
Iomeprol	C_17_H_22_I_3_N_3_O_8_	777.089	Non-ionic	0.107	261–262	Low
Iopromide	C_18_H_24_I_3_N_3_O_8_	791.116	Non-ionic	0.796	174–180	Low

**Table 2:** Range of concentrations of iodine contrast agents in Europe [11,20,46,47].

	Concentrations (μg L^−1^)			
	Diatrizoate	Iohexol	Iopamidol	Iomeprol
Hospital wastewater	17.1–61	0.07–3810	0.03–2599	0.05–2400
Ground water	0.02–9.6	0.003–0.187	0.006–0.47	0.003–1.655
Surface water	0.032–4.55	0.01–1.326	0.008–3.2	0.023–6.1
Drinking water	0.0009–1.2	0.001–0.034	0.02–0.27	0.0013–0.034

**Table 3:** Examples of AOPs for ICM decomposition (**A**) [10,48,56,69,74] and summary of main available methods with remarks on kinetic mechanism (**B**) [80,81,82,83,84].

(A)						
ICM Degradation Process	Experimental Conditions	Results	Intermediate Products	Toxicity Monitoring	Advantages	Disadvantages
UV Photolysis and UV/TiO_2_ Photocatalysis	UV low pressure lamp, TiO_2_: 500 mg/L, pH: 7, matrix: model solution, time 60–120 min	ICM removal without TiO_2_: iodipamide 29%, iohexol: 27%, diatrizoate: 30%, removal with TiO_2_: iodipamide 28%, iohexol 38%, diatrizoate 40%	They contained partially deiodinated structures with transformed aromatic rings and hydrophilic groupings	Toxicity identification has not been carried out	Capable of degrading a wide range of organic micropollutants; UV/TiO_2_ photocatalysis enables the generation of hydroxyl radicals (·OH); TiO_2_ is chemically stable, inexpensive, and non-toxic.	Direct UV photolysis is often ineffective for persistent compounds such as iodinated contrast media (ICM); Limited light penetration in turbid or organic-rich waters; Electron–hole recombination in TiO_2_ reduces process efficiency.
UV/H_2_O_2_ and UV/H_2_O_2_/Fe^2+^ processes (Fenton, photo-Fenton)	Medium pressure UV lamp, H_2_O_2_: 10–50 mg/L, Fe^2+^: 1–5 mg/L, pH: 3.5–6, matrix: distilled water, wastewater, urine, time up to 60 min	Removal of iohexol in distilled water > 90%, wastewater about 60%.	Carboxylic acid derivatives and aromatic amines formed by partial degradation of the ring structure and functional groups of iohexol have been identified	Toxicity identification was carried out using the Microtox test—luminescent bacteria *Aliivibrio fischeri*—by LC-MS/MS	Highly efficient generation of hydroxyl radicals; Effective degradation of persistent pharmaceuticals and iodinated compounds; Photo-Fenton enhances degradation rates and reduces reagent consumption.	High consumption of hydrogen peroxide; Classical Fenton reactions require acidic conditions (pH ≈ 2.5–3); Formation of iron-containing sludge requiring disposal.
UV/S_2_O_8_^2−^	UV lamp, S_2_O_8_^2−^: 10–100 mg/L, pH: 5–7, temperature: 25 °C, matrix: distilled water, time: up to 90 min.	100% degradation of iopromide, 90% mineralization of iohexol were obtained	Toxic CHI_3_ was created	Toxicity was monitored with *Vibrio fischeri* and *Daphnia magna*—using LC-QTOF MS/MS	Generation of sulfate radicals (SO_4_^−^), which are strong and more selective oxidants than ·OH; Effective for compounds resistant to hydroxyl radical oxidation; Operates over a broader pH range.	High cost of persulfate reagents; Potential increase in sulfate concentration in treated water; Often incomplete mineralization and accumulation of transformation products.
Ozonation and reductive dehalogenation	O_3_: 1–3 mg/L, pH: 7 (ozonation), pH: 2–3 (reductive dehalogenation), matrix: hospital wastewater, urine, time up to 60 min	Degradation of iopromide, diatrizoate 60–80%	Iodinated phenols, aldehydes, carboxylic acids were formed	No complete analysis—monitored by GC-MS and HPLC	Rapid reaction kinetics with many pharmaceuticals; Effective cleavage of aromatic structures and carbon–iodine bonds; Easily integrated into existing water treatment infrastructure.	Selective reactivity; some compounds react slowly with ozone; Formation of toxic by-products, including iodinated DBPs; High energy and capital costs.
Electrochemical reduction and electrochemical oxidation	Cathode: graphite felt/Pd Anode: boron doped diamond, pH: 7–8.5, current: 10–50 mA/cm^2^, matrix: model water, wastewater, time: 120–240 min	Total deiodination of diatrizoate, mineralization of intermediates > 95%	3,5-diacetamidobenzene acid was formed	Monitoring by HPLC and with *Vibrio fischeri* and *Daphnia magna*	In situ generation of hydroxyl radicals without chemical additives; High efficiency for persistent and recalcitrant contaminants; Easily automated and precisely controlled.	High energy demand; Electrode degradation (e.g., boron-doped diamond electrodes); Formation of chlorinated by-products in chloride-containing waters.
**(B)**						
**Method**	**Mechanism**		**Oxidative vs. Reductive**		**Mineralization**	
UV Photolysis (direct UV)	Direct photolytic excitation → bond cleavage and radical formation		Primarily oxidative/direct photolysis		Partial removal: e.g., ~27–30% decay for iohexol/diatrizoate via UV alone (not full mineralization)	
UV/TiO_2_ Photocatalysis	UV excitation of TiO_2_ → e^−^/h^+^ → ∙OH and oxidative radical formation		Oxidative (photocatalytic oxidation)		Partial degradation but generally low mineralization of ICM alone; improves biodegradability	
UV/H_2_O_2_ Advanced Oxidation	UV splits H_2_O_2_ → 2 × ∙OH radicals		Oxidative (AOP)		Often increases degradation vs. UV alone; specific ICM mineralization % under UV/H_2_O_2_ alone rarely reported but TOC/complete mineralization is limited unless optimized; requires ideal conditions.	
UV/H_2_O_2_/Fe^2+^ (Photo-Fenton)	UV + H_2_O_2_ + Fe^2+^ → enhanced ∙OH production		Oxidative (photo-Fenton AOP)		Photo-Fenton improves overall oxidation and can improve mineralization relative to H_2_O_2_ only; mineralization strongly depends on conditions (pH, Fe^2+^, etc.); reported mineralization up to ~64.7% for diatrizoate (in related studies)	
UV/S_2_O_8_^2−^ (UV/Peroxydisulfate)	UV generates sulfate radicals (SO_4_∙^−^) + some ∙OH		Oxidative (AOP)		Can achieve rapid degradation and near-complete mineralization under optimal conditions (e.g., complete iopromide removal, near-complete TOC loss within ~80 min, dependent on oxidant dosing)	
Ozonation (O_3_)	Ozone oxidation + radical formation (e.g., ∙OH)		Oxidative		Fast parent compound loss but incomplete mineralization; produces oxidation products and inorganic iodate; organics remain partially transformed	
Reductive Dehalogenation (e.g., zero-valent metals, specialized reduced media)	Electron-driven removal of I from aromatic rings		Reductive		Reductive deiodination removes iodine substituents → simpler compounds which may be more biodegradable; does not fully mineralize carbon skeleton unless followed by oxidative/bio step	
Electrochemical Oxidation (Anodic)	High-potential oxidation at anode produces ∙OH and other oxidants		Oxidative		High degradation; mineralization varies ~60–80% DOC reduction in some reports after prolonged treatment	
Electrochemical Reduction (Cathodic)	Electron addition at cathode supports deiodination and reductive break-down		Reductive		Deiodination improves biodegradability and partial transformation; does not inherently fully mineralize organics	
Electrochemical Reduction + Oxidation (Sequential)	First reductive deiodination, then oxidative mineralization		Reductive then Oxidative		Combined approach improves biodegradability and overall mineralization yields vs. single steps; mineralization can increase significantly vs. reduction alone	

## Data Availability

No new data were created or analyzed in this study. Data sharing is not applicable to this article.
